# TLR9 Promoter Polymorphism Is Associated with Both an Increased Susceptibility to Gastric Carcinoma and Poor Prognosis

**DOI:** 10.1371/journal.pone.0065731

**Published:** 2013-06-12

**Authors:** Xiaoyong Wang, Lening Xue, Yang Yang, Lijuan Xu, Guoxin Zhang

**Affiliations:** 1 Department of Gastroenterology, Changzhou No. 2 Hospital, Affiliated with Nanjing Medical University, Changzhou, China; 2 Department of Gastroenterology, The First Affiliated Hospital of Nanjing Medical University, Nanjing, China; University of Montana, United States of America

## Abstract

**Objective:**

Genetic polymorphisms of Toll-like receptors (TLRs) may influence the effects of *H. pylori* infection and play important roles in gastric carcinogenesis. The aim of this study was to determine whether the polymorphisms of TLR4 and TLR9 are associated with susceptibility to gastric carcinoma and its prognosis.

**Methods:**

This study consisted of 314 patients with gastric cancer and 314 healthy controls. The polymorphisms were assessed using polymerase chain reaction–restriction fragment length polymorphism (PCR–RFLP) analysis. Survival was analyzed by Kaplan–Meier survival curves.

**Results:**

No variant genotypes of TLR4+896A/G, TLR4+1196C/T, or TLR9 -1237T/C were detected. For TLR9 -1486 T/C, multiple logistic regression analyses revealed that compared with the TT homozygote, patients with both the TC variant (adjusted odds ratio (OR) = 1.47, 95% confidence interval (CI) = 1.04–2.10) and the CC variant (adjusted OR = 1.63, 95% CI = 1.01–2.64) had higher risks of gastric cancer. Further stratification analyses revealed that an increased risk of gastric cancer associated with C carriers was evident among females (adjusted OR = 1.84, 95%CI = 1.02–3.33), in younger subjects aged less than 60 years old (adjusted OR = 1.86, 95%CI = 1.15–3.00), and subjects with *H. pylori* infection (adjusted OR = 1.53, 95% CI = 1.03–2.27). We also observed a significant association between C carriers and noncardia gastric cancer (adjusted OR = 1.51, 95% CI = 1.03–2.20). In addition, we demonstrated that the C carrier genotype and *H. pylori* infection may have a synergistic effect and conferred an OR of 2.44 for developing gastric cancer. TLR9 -1486C was also identified as an independent marker of poor survival of carcinoma.

**Conclusions:**

Our results suggest that TLR9 -1486C carriers are associated with an increased risk and poor prognosis of gastric carcinoma in the Chinese population.

## Introduction

*Helicobacter pylori* is a major cause of gastric cancer [1]. However, only a small percentage of *H. pylori-*infected patients actually develop gastric cancer. Hence, gastric tumorigenesis is multifactorial; the genetic composition of the infecting strains and the genetic make-up of the host, especially the variations in the host immune responses, are contributing factors [2]. Lichtenstein et al. reported that exposures to exogenous risk factors may contribute to a 72% increased risk of developing gastric cancer, and the host susceptibility may also play an important role in 28% of gastric cancer development [3].

The host immune response has a strong role in defining the outcome of *H. pylori* infection, and polymorphisms in genes that control this immune response have been shown to influence the risk of gastric cancer[4]–[6]. Toll-like receptors (TLRs) are important innate immunity regulators that can be activated upon recognition of bacterial and viral ligands, known as pathogen-associated molecular patterns (PAMPs)[7]–[8], and provide the first line of host defense against several conditions including *H. pylori* infection [9]. TLRs are characterized by the presence of an extracellular leucine-rich domain and an intracellular Toll/interleukin (IL)-1 receptor (TIR) domain [10]. To date, 13 related TLR genes have been identified and characterized (TLR1–TLR13) [11]. TLR4 and TLR9 are known to be expressed by gastric epithelial cells in the human stomach[12]–[13], which play an important role in innate immune signaling to *H. pylori* [14]–[16]. Some studies have reported controversial findings surrounding the role of TLR4 in activation of the innate immune response to *H. pylori* [17]–[18]. However, most studies have concluded that the TLR4/MD-2 system is a potent receptor complex involved in the response to *H. pylori* lipopolysaccharides in the stomach[14]–[15], [19], with subsequent activation of NF-κB and production of proinflammatory cytokines [8], [20]. TLR9 recognizes unmethylated CpG oligonucleotides that are abundant in bacterial DNA, leading to NF-κB activation[21]–[22].

TLR activation plays a role in initiating the inflammatory response. Consequently, dysregulation of TLR signaling may contribute to an unbalanced ratio between pro- and anti-inflammatory cytokines and thus lead to a higher risk of developing chronic inflammatory diseases and cancer [9]. It is well known that chronic inflammation plays an important role in promoting gastric cancer[23]–[24]. Furthermore, the association between an IL-1 receptor (IL-1R) gene polymorphism and enhanced gastric cancer risk has been demonstrated[25]–[26]. Of note, TLRs are characterized by a TIR intracellular domain with high homology to IL-1R. For all these reasons, TLR polymorphisms should be studied as possible susceptibility factors in the development of gastric cancer.

Polymorphisms in TLR4 have already been studied. Among them are +896A/G (SNP ID: rs 4986790) and +1196C/T (SNP ID: rs 4986791) in exon 3. These two nonsynonymous single nucleotide polymorphisms (SNPs) affect the TLR4 extracellular domain and result in amino acid exchanges: an aspartic acid for a glycine at position 299 (Asp299Gly) and a threonine for an isoleucine at position 399 (Thr399Ile) [27]. Worldwide, studies have shown inconsistent results regarding the association between TLR4 polymorphisms and gastric cancer [6], [24], [28].

Several SNPs have been identified within the TLR9 gene. The -1237T/C SNP (rs5743836) and the -1486T/C SNP (rs187084), located in the promoter region, alter the functional ability of TLR9 [29]. The variant alleles of these polymorphisms can possibly modify the response to *H. pylori* infection, thereby increasing the risk of gastric cancer.

The aims of the present work were to investigate the potential functional polymorphisms and how they relate to the risk of gastric cancer. We also investigated the association between TLR polymorphisms and the risk of *H. pylori* infection. In addition, we evaluated whether TLR polymorphisms affect the overall survival of gastric cancer patients, either independently or through their association with tumor characteristics of clinical importance.

## Materials and Methods

### Ethics Statement

This study was approved by the Ethics Committee of Nanjing Medical University. Written informed consents were obtained from all subjects before participating in the study.

### Study Population

This study consisted of 314 patients with gastric cancer and 314 healthy controls. All subjects were unrelated ethnic Han Chinese and residents in Jiangsu Province, People’s Republic of China. Patients were consecutively recruited between July 2007 and July 2009, from Changzhou No. 2 Hospital, Affiliated with Nanjing Medical University and the First Affiliated Hospital of Nanjing Medical University. All cases were histologically confirmed as having adenocarcinoma of the stomach, and were classified into diffuse type, intestinal type and mixed type according to the Lauren classification by pathologists in participating hospitals. These patients were divided into two subgroups according to the location of the tumor: cardia cancer and noncardia cancers. We defined cardia cancer as a lesion primarily involving the area between 1.0 cm proximal and 2.0 cm distal to the gastroesophageal junction, and we classified lesions primarily involving the fundus, body, or antrum as noncardia cancers. The population controls were selected from cancer-free individuals living in the same residential areas as the cases and were frequency matched to the cases based on age (±5 years) and sex. The median duration of follow-up was 35 months (ranging from 3 to 56 months). Clinical data of patients were collected from medical records and structured patient interviews using a questionnaire. Fifty-three of the patients were transferred to other hospitals with their medical history records or were unwilling to complete the questionnaires; in addition, some clinical data of patients were unable to be traced, leaving a total of 261 patients with sufficient clinical information available. After the interview, 2 mL of venous blood was collected from each subject.

### Serological Determination of *H. pylori* Infection

An indirect solid-phase immunochromatographic assay was used to investigate the presence of immunoglobulin G (IgG) antibodies to *H. pylori* (Genelabs Diagnostics Pty Ltd., Singapore). This method was validated in our lab previously with an accuracy of 92.3% [30]. The test was performed in accordance with the manufacturer’s instructions. The absence of the “A” band, which is a control line, indicated an invalid result. The presence of an ‘‘A B C’’ band or an ‘‘A C’’ band was categorized as positive for *H. pylori* infection.

### Genotype Analysis

Genomic DNA was extracted from a leukocyte pellet by proteinase K digestion followed by phenol–chloroform extraction and ethanol precipitation. The TLR4+896A/G, TLR4+1196C/T, and TLR9 -1237T/C polymorphisms were assessed using polymerase chain reaction-restriction fragment length polymorphism (PCR-RFLP) analysis as described previously[6], [31]–[32]. TLR9 -1486T/C was also genotyped by PCR-RFLP. In brief, the primers of TLR9 -1486T/C were 5′-TCCCAGCAGCAACAATTCATTA-3′ (forward) and 5′-CTGCTTGCAGTTGACTGTGT-3′ (reverse), which generated a 499-bp fragment. The 20-µL PCR mixture contained 50 ng of genomic DNA, 12.5 pmol of each primer, 0.1 mM each deoxynucleotide triphosphate, 1× PCR buffer, 1.5 mM MgCl_2_, and 1.0 U of Taq polymerase. The PCR profile for detecting the TLR9 -1486T/C polymorphism consisted of an initial melting step of 95°C for 4 min; 35 cycles of 95°C for 30 s, 60°C for 20 s, and 72°C for 30 s; and a final extension step of 72°C for 5 min. The PCR product was then digested with AflII (New England Biolabs, Beverly, MA, USA). The digested products were visualized on a 3.0% agarose gel containing ethidium bromide. The products that could be cleaved showed two bands at 172 and 327 bp, representing allele T, whereas those that could not be cleaved had a single 499 bp band representing allele C. Genotyping was performed without knowing the status (case or control) of the subjects. To confirm the genotype ascribed by RFLP, 110 individuals were included in a quality control study, and the PCR products were subjected to direct sequencing. The results of the two methods were in agreement.

### Statistical Analysis

Hardy–Weinberg equilibrium of alleles was assessed using chi-squared (χ^2^) analysis. For each characteristic including genotype frequencies, the differences in the distribution between cases and controls were tested using the chi-squared test. The association between the polymorphisms and gastric cancer risk was estimated using an odds ratio (OR) and a 95% confidence interval (CI). Logistic regression was used to control for potential confounders (sex, age, and *H. pylori* serology) and to estimate crude and adjusted ORs and 95% CIs. Kaplan–Meier survival curves and the log–rank test for trend were used to evaluate the relationship between TLR polymorphisms and the prognosis from the date of primary diagnosis to the end of follow-up. Multivariate Cox regression analysis was performed to assess the prognostic value of the TLR polymorphism with adjustments for age, gender, *H. pylori* serology, and tumor, lymph node, and metastasis (TNM) stage. Hazard ratios (HRs) and 95% CIs were calculated from the Cox regression model. Differences were regarded significant at *P*<0.05. All data analyses were performed using SPSS software (version 13.0; Chicago, IL, USA).

## Results

### Study Population Characteristics

The cancer cases and controls seemed to be adequately matched in terms of sex and age. The median age was 60 years old (range, 21–84 years) for the patients and 59 years old (range, 22–85 years) for the controls (*P* = 0.44). There was no difference between patients and controls in terms of sex distribution (67.2% men in cases vs. 67.2% in controls; *P* = 1.00). The percentage of subjects who tested positive for *H. pylori* infection was significantly higher in the case group than in the control group (*P* = 0.02). In addition, *H. pylori* infection increased the risk of developing gastric cancer (OR = 1.51, 95%CI = 1.07–2.13). The characteristics of the subjects are summarized in Table 1.

**Table 1:** Baseline characteristics of cases and controls.

Characteristics	Cases	Controls	*P* value
	(n = 314)	(n = 314)	
Age (years)			0.44
Median	60	59	
Standard deviation	12.61	12.01	
Range	21–85	22–85	
Sex			1.00
Male	211 (67.20%)	211 (67.20%)	
Female	103 (32.80%)	103 (32.80%)	
*H. pylori*			0.02
Seronegative	81 (25.80%)	108 (34.39%)	
Seropositive	233 (74.20%)	206 (65.61%)	
Location			
Cardia	87 (27.71%)		
Noncardia	208 (66.24%)		
TNM stage			
I	53 (20.31%)		
II	37 (14.18%)		
III	111 (42.53%)		
IV	60 (22.99%)		
Follow-up, months			
Median	35		
Range	3–56		

TNM: Tumor, Lymph Node, Metastasis.

Among 314 patients, 53 have missing TNM stage data.

### Prevalence of TLR4+896A/G, TLR4+1196C/T, and TLR9 -1237T/C in the Chinese Population

TLR4+896A/G, TLR4+1196C/T, and TLR9 -1237T/C were typed in all 628 subjects. However, in this study, all included individuals had the same genotype: TLR4+896AA, TLR4+1196CC, and TLR9 -1237TT. Thus, there were no TLR4+896A/G, TLR4+1196C/T, or TLR9 -1237T/C polymorphisms among the ethnic Chinese examined.

### Association of the TLR9 -1486T/C Polymorphism with Risks of *H. pylori* Infection

In our study population, the alleles at TLR9 -1486 loci were in Hardy–Weinberg equilibrium (*P = *0.93). We investigated the association between the TLR9 **-**1486T/C polymorphism and *H. pylori* infection risk in gastric cancer patients as well as in the control group. In the gastric cancer patients, the TLR9 genotype distribution was 31.33% TT, 51.50% TC, and 17.17% CC in *H. pylori-*positive patients; and 25.93% TT, 54.32% TC, and 19.75% CC in *H. pylori-*negative patients. Meanwhile, in the control group, the TLR9 genotype distribution was 40.29% TT, 44.66% TC, and 15.05% CC in *H. pylori*-positive subjects; and 36.11% TT, 51.85% TC, and 12.04% CC in *H. pylori-*negative subjects. No association was observed between the TLR9 genotype and the risk of *H. pylori* infection (*P = *0.64 and 0.46, respectively, by 3×2 tables using the χ^2^-test).

### TLR9 -1486 C Carriers were Associated with an Increased Risk of Gastric Cancer

The genotype distributions of TLR9 -1486 T/C in the cases and the controls are shown in Table 2. Compared with the TT homozygote, the C heterozygote (TC) (adjusted OR = 1.47, *P = *0.03, 95% CI = 1.04–2.10) and homozygote (CC) (adjusted OR = 1.63, *P = *0.04, 95% CI = 1.01–2.64) both had significantly higher gastric cancer risks (Table 2), suggesting a dominant effect of the C allele. Overall, C carriers (either TC or CC) were associated with a higher gastric cancer risk when compared with TT carriers (adjusted OR = 1.51, *P = *0.02, 95% CI = 1.08–2.11).

**Table 2:** The genotype distributions of TLR9-1486 T/C in cases and controls.

Genotype	Controls (314)		Patients (314)		Crude OR	Gastric Carcinoma				
	No.	%	No.	%		95% CI	*P*	Adjusted OR^a^	95% CI	*P*
TT	122	38.9%	94	29.9%	1.00					
TC	148	47.1%	164	52.2%	1.44	1.02–2.04	0.04	1.47	1.04–2.10	0.03
CC	44	14.1%	56	17.8%	1.65	1.02–2.66	0.04	1.63	1.01–2.64	0.04
Recessive model										
Others	270	85.9%	258	82.2%	1.00					
CC	44	14.1%	56	17.8%	1.33	0.87–2.05	0.19	1.33	0.86–2.05	0.20
Dominant model										
TT	122	38.9%	94	29.9%	1.00					
Others	192	61.1%	220	70.1%	1.49	1.07–2.07	0.02	1.51	1.08–2.11	0.02

a Adjusted for age, sex, and *H. pylori* infection.

OR, odds ratio; CI, confidence interval.

### Stratification Analyses of TLR9 -1486T/C and the Risk of Gastric Cancer

Stratified analyses based on the dominant models are shown in Table 3. A significantly increased risk of gastric cancer associated with C carriers was evident among females (adjusted OR = 1.84, 95%CI = 1.02–3.33), subjects with *H. pylori* infection (adjusted OR = 1.53, 95% CI = 1.03–2.27), and noncardia gastric cancer patients (adjusted OR = 1.51, 95% CI = 1.03–2.20), compared with the TLR9 -1486 TT genotypes. When stratified by the histologic subtype and tumor depth, the C carriers were found to be positively associated with intestinal gastric cancer (adjusted OR = 1.79, 95% CI = 1.93–2.69) and advanced gastric cancer (adjusted OR = 2.33, 95% CI = 1.43–3.80), compared with the TT genotype (Table 3). We evaluated the role of the TLR9 -1486T/C polymorphism in gastric cancer in different age groups. In those less than 60 years old, carriers of the C allele were significantly associated with gastric cancer with an OR of 1.86 (95% CI = 1.15–3.00), compared with those with the TT genotype. As shown in Table 4, patients who were C carriers with *H. pylori* infection (adjusted OR = 2.44, 95% CI = 1.36–4.36) were at a significantly increased risk of gastric carcinoma, compared with those with the TT genotype without *H. pylori* infection. However, because of the limited study sample size, all the results from stratified analyses were preliminary.

**Table 3:** Stratified analyses between TLR9 -1486 T/C polymorphisms and gastric cancer risk.

variable	T-1486C (case/control)		OR (95% CI)*		*P*
	TT	TC+CC	TT	TC+CC	
Age			1.00		
<60	47/69	109/99	1.00	1.86 (1.15–3.00)	0.01
≥60	47/53	111/93	1.00	1.36 (0.84–2.19)	0.22
Sex			1.00		
Male	61/78	150/133	1.00	1.44 (0.95–2.16)	0.08
Female	33/44	70/59	1.00	1.84 (1.02–3.33)	0.04
Hp infection			1.00		
Negative	21/39	60/69	1.00	1.67 (0.87–3.22)	0.12
Positive	73/83	160/123	1.00	1.53 (1.03–2.27)	0.03
Location site					
Cardia	27/122	60/192	1.00	1.15 (0.70–1.90)	0.58
Non-cardia	63/122	145/192	1.00	1.51 (1.03–2.20)	0.03
Lauren’s					
Intestinal	47/122	132/192	1.00	1.79 (1.93–2.69)	0.01
Diffuse	24/122	58/192	1.00	1.59 (0.94–2.71)	0.09
Tumor depth					
Early	18/122	30/192	1.00	1.15(0.61–2.17)	0.68
Advanced	27/122	98/192	1.00	2.33(1.43–3.80)	0.01

* Adjusted for age, sex, and *H. pylori* infection in a logistic dominant model.

OR, odds ratio; CI, confidence interval.

**Table 4:** Association between TLR9 -1486 T/C and gastric carcinoma in relation to *H. pylori* infection.

TLR9 -1486 T/C	*H. pylori* infection	Odds ratio* (95% Confidence interval)	*P* value
TT	Negative	1.00	
TT	Positive	1.61 (0.86–2.99)	0.13
TC+CC	Negative	1.67 (0.87–3.22)	0.12
TC+CC	Positive	2.44 (1.36–4.36)	<0.001

* Adjusted for age and sex.

### TLR9 Genotype and Disease Progression

The overall survival of gastric cancer patients was analyzed for dependence on TLR9 -1486 genotypes using Kaplan-Meier survival curves. Kaplan–Meier survival curves showed that overall survival of gastric cancer patients was associated with the TLR9 -1486 genotypes. There was a statistical difference among different TLR9 -1486 genotypes. Cox proportional hazards analysis with adjustments for age, sex, *H. pylori* infection, and TNM stage showed that TC (HR = 2.49, CI = 1.48–4.17, *P*<0.001) and CC (HR = 4.95, CI = 2.87–8.55, *P*<0.001) genotypes were significantly associated with poor survival, compared with the TT genotype. High TNM stage including III (HR = 3.91, CI = 1.86–8.23, *P*<0.001) and IV (HR = 10.04, CI = 4.70–21.45, *P*<0.001) certainly showed a strong association of poor overall survival, compared with stage I.

## Discussion

In this study, we did not detect TLR4+896G, TLR4+1196T, or TLR9 -1237C alleles in gastric cancer patients and healthy controls in the Chinese population. All 628 subjects had the same genotype: TLR4+896AA, TLR4+1196CC, and TLR9 -1237TT. These results were similar to those found by Hang et al. who screened 491 Chinese textile workers in Shanghai and did not detect alleles +896G or +1196T of TLR4 [33]. Similarly, Ng et al. screened 799 healthy Chinese people and 467 systemic lupus erythematosus (SLE) patients in Hong Kong for TLR9 -1237T/C polymorphisms, and they found that only 12 people were C carriers [34]. Similar results have also been reported in other Japanese and Korean studies[28], [35]–[36]. TLR4+896G, TLR4+1196T, and TLR9 -1237C alleles may be very rare, at least in Chinese, Korean, and Japanese populations; thus, this gene may not be a candidate for determining gastric cancer susceptibility in this area. Our results are in contrast with those previously reported in several Caucasian populations that identified that TLR4+896G and TLR4+1196T alleles increased the risk of gastric carcinoma [6], [37]. The reason for the differences between these reported results are probably due to racial differences.

In the present study, we found that the TLR9 -1486T/C polymorphism is associated with an increased risk of gastric cancer in the Chinese population. The precise mechanism of this phenomenon is unclear, although *in silico* analysis has shown that this SNP creates a putative Sp1 binding site, which may be functionally relevant [38]. In addition, an association between the C allele of TLR9- 1486T/C and an increased risk of SLE in the Japanese population has been found; and a reporter gene assay showed that this allele downregulates TLR9 expression [39]. Because regulation of TLR9 expression and signaling play important roles with respect to the immune response against *H. pylori* [40], [41], this altered function may be relevant in carcinogenesis of the stomach. To date, there are no functional data available for the TLR9 -1486T/C polymorphism with regard to gastric cancer. We intend to assess TLR9 expression in gastric epithelial cells by immunohistochemistry and real-time quantitative PCR experiments and determine its correlation with the genetic polymorphisms. We also intend to assess the functional modulation of human gastric epithelial cells in primary cultures by incubation with CpG oligodeoxynucleotides to investigate whether differential signaling and cellular responses occur in patients expressing different TLR9 SNPs.

Our results are inconsistent with the study conducted in another Chinese population that demonstrated that the TLR9 -1486T/C polymorphism was not associated with an increased gastric cancer risk [42]. The reason for these different findings remains unclear, which might due to heterogeneous genetic backgrounds. Our study conducted in Nanjing and Changzhou counties, two areas in Jiangsu province, southern China, however, Zeng HM *et al*. conducted the study in Linqu county, Shangdong province, northern China. Heterogeneous genetic backgrounds in different populations is likely to explain the discrepancy in the results.

Considering the potential functional relevance between TLR9 and *H. pylori* infection, we were also interested in evaluating the possible interaction between the TLR9 polymorphism and *H. pylori* infection. In our study, we observed significant joint effects of the TLR9 -1486T/C polymorphism and *H. pylori* infection, suggesting a potential gene–environment interaction. The interaction between *H. pylori* infection and the presence of the C allele conferred an OR of >2.0 for developing gastric cancer. The molecular mechanisms underlying this phenomenon may be as follows. *H. pylori* is an established etiologic factor for gastric cancer and has been classified by the World Health Organization (WHO) as a Class I human carcinogen. TLR9 is an important *H pylori*-recognizing receptor [40]. TLR9 expression and signaling is quite important in *H. pylori*-associated gastric cancer [43]. In addition, appropriate activation of Toll-like receptors has been shown to be critical for the protection and recovery from gut injury [44]. A study has shown that the C allele of TLR9 -1486T/C downregulates TLR9 expression [39], which may affect *H. pylori* recognition. Thus, deficient *H. pylori* recognition and signaling via TLRs may reduce the innate immune response during the acute phase of infection and favor persistent infection; it might also lead to an inefficient stimulation of the adaptive immune response during chronic infection. Furthermore, it is well known that chronic inflammation plays an important role in promoting gastric cancer. Therefore, individuals with both CC or TC genotypes and *H. pylori* infection were expected to have the highest risk of gastric cancer.

In the stratification analysis, we found that the TLR9 -1486T/C polymorphism was associated with an increased risk of gastric cancer among subgroups of younger subjects (age <60 years old) but not older subjects. As a person ages, a large amount of DNA damage and genomic alterations accumulate in the body, which would facilitate carcinogenesis [45]. Besides, deleterious alterations also occur to the immune response with aging [46], which further promote cancer development. It has been widely accepted that age is the most important risk factor for cancer. Therefore, the increased risk associated with being a C carrier in older subjects may be overwhelmed by the presence of more genomic aberrations and the deleterious alterations of the immune system in them, which may partly contribute to the age difference we observed. We also found that the C carriers were positively associated with intestinal gastric cancer. The intestinal gastric cancer develops in stomachs affected by chronic inflammation with passing through the intermediate steps of atrophic gastritis or intestinal metaplasia. The C allele of TLR9 -1486T/C downregulates TLR9 expression [39], which may affect *H. pylori* recognition. Thus, this polymorphism may lead to persistent infection. Therefore, C carriers increase the risk for the development of intestinal-type gastric cancer. When stratified by sex, the association was restricted to females, but there is the possibility that this finding may be attributed to chance due to the limited numbers of patients.

The TNM stage, which is a widely accepted prognostic factor, significantly correlated with the survival of gastric cancer patients in our study. We also found that the TLR9 -1486C allele was an independent prognostic factor by Cox proportional hazard analysis of survival in this study. The molecular mechanism of this phenomenon is unclear, and further studies concerning the TLR9 -1486C allele and poor prognosis are needed.

Some limitations in our study need to be addressed. First, inherent selection bias cannot be completely excluded. The cancer cases are from two hospitals and may not be representative of the entire target population. However, we reduced the selection bias to the lowest possible level by matching the age, residential areas, and gender between the cases and controls. Second, alcohol and tobacco smoking are well-known causes of gastric cancer. Genetic polymorphisms may interact with these factors to influence gastric caner risk. Unfortunately, we did not have available information on these factors in our study, and therefore we were unable to perform further analysis.

We investigated the TLR9 -1486T/C polymorphism in a limited region of China. Because the polymorphism may show variations in different ethnic groups, further studies are needed in a larger and ethnically diverse population to confirm the impact of this gene on the susceptibility of gastric cancer.
